# A novel self-shaping magnetic compression anastomosis ring for treatment of colonic stenosis

**DOI:** 10.1055/a-2183-8942

**Published:** 2023-10-24

**Authors:** Miaomiao Zhang, Shuixiang He, Huanchen Sha, Hairong Xue, Yi Lv, Xiaopeng Yan

**Affiliations:** 1 Department of Hepatobiliary Surgery, The First Affiliated Hospital of Xi’an Jiaotong University, Xi’an, Shaanxi, China; 2 National and Local Joint Engineering Research Center of Precision Surgery & Regenerative Medicine, The First Affiliated Hospital of Xi’an Jiaotong University, Xi’an, Shaanxi, China; 3 Department of Gastroenterology, The First Affiliated Hospital of Xi’an Jiaotong University, Xi’an, Shaanxi, China

Magnetic compression anastomosis (MCA) has been used for the treatment of severe colorectal stenosis and atresia 1 2 3 . Herein we report a case of stenosis of the descending colon treated with transanal single-channel MCA.

A 15-year-old boy had severe stenosis of the descending colon due to repeated pancreatitis. Colonoscopy and colonography showed long, severe, and eccentric colonic stenosis ( Fig. 1 ). After obtaining consent from the patient’s mother, we performed endoscopy-assisted MCA in the descending colon using a novel self-shaping magnetic anastomosis ring, designed by ourselves ( Video 1 ). The surgical plan is illustrated in Fig. 2 .

**Fig. 1:**
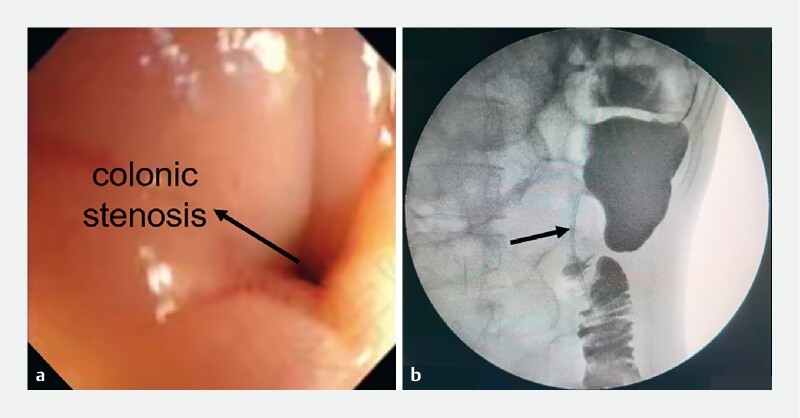
Severe eccentric colonic stenosis in a 15-year-old boy on: **a** colonoscopy and **b** colonography.

**Video 1** Self-shaping ring for magnetic compression anastomosis (MCA), and use for the treatment of colonic stenosis.


**Fig. 2:**
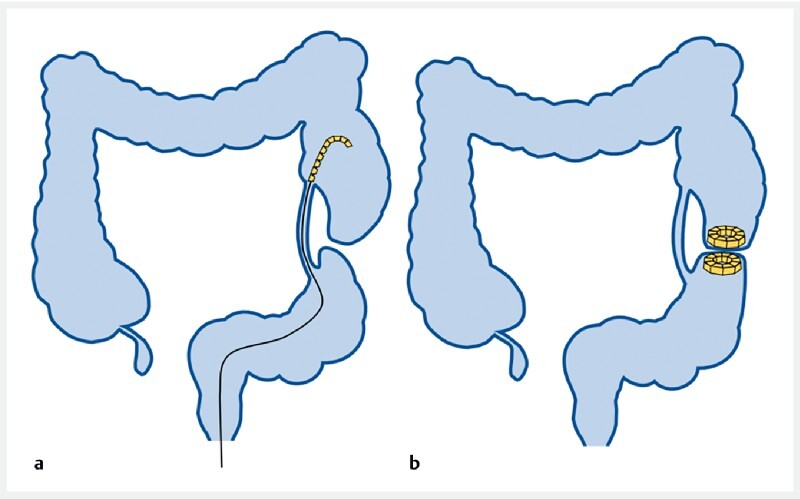
Surgical plan. **a** Placement of the self-shaping magnetic anastomosis ring in the colon. **b** Bypass magnetic compression anastomosis was established due to the attraction between daughter and parent magnetic rings.

Under X-ray surveillance, a stiff guidewire was inserted through the narrow segment of the colon into the proximal colon using a colonoscope. The 10 units of the novel self-shaping magnetic ring were inserted along the guidewire in a linear fashion (see Video 1 ). Next, under X-ray surveillance, the catheter was slowly passed along the guidewire to insert all the linear magnetic units into the proximal end of the colonic stenosis. The push tube was fixed, and the guidewire was slowly removed. X-ray showed that the adjacent magnetic units were gradually and successively attracted to each other, leading to the formation of a ring; this constituted the daughter magnet. Two more series of magnetic units were looped into rings and fixed together using nylon wire; these served as the parent magnet. The colonoscope was used to navigate the parent magnet to the distal end of the colonic stenosis segment through the anus ( Fig. 3 a ). The position of the parent magnet was adjusted under X-ray guidance to enable its attraction to the daughter magnet ( Fig. 3 b ). On day 7 after the operation, the daughter and parent magnets were expelled through the anus ( Fig. 4 a ). Colonoscopy was performed immediately afterward ( Fig. 4 b , Video 1 ). The patient has been followed up for 9 months and continues to maintain good health.

**Fig. 3:**
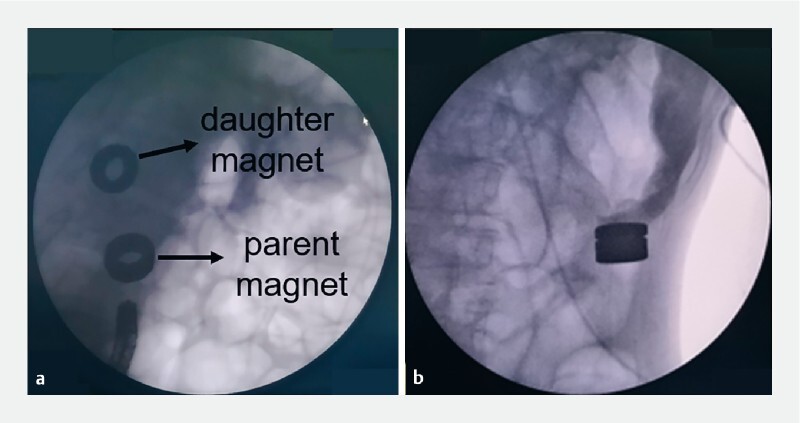
X-ray view of the daughter and parent magnets: **a** located at the opposite ends of the narrow colon; **b** when attracted to each other.

**Fig. 4:**
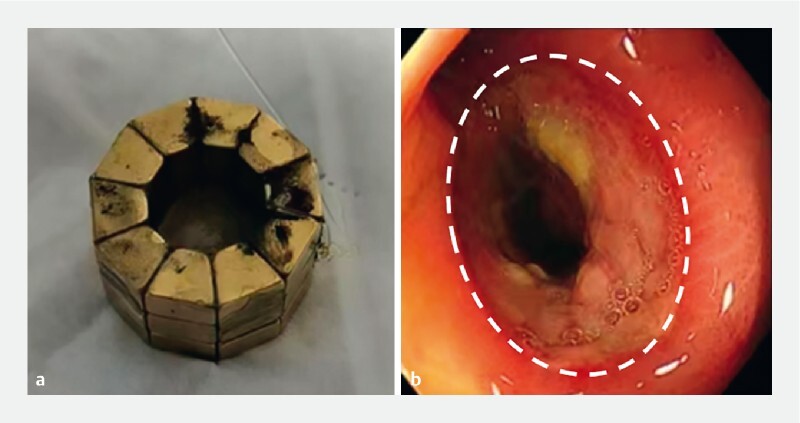
Postoperative day 7: **a** the self-shaping magnetic anastomosis rings were expelled; **b** colonoscopic view of the anastomosis

Endoscopy_UCTN_Code_TTT_1AQ_2AF

## Notes

Endoscopy E-Videos

https://eref.thieme.de/e-videos
*E-Videos* is an open access online section of the journal *Endoscopy* , reporting on interesting cases and new techniques in gastroenterological endoscopy. All papers include a high-quality video and are published with a Creative Commons CC-BY license. Endoscopy E-Videos qualify for HINARI discounts and waivers and eligibility is automatically checked during the submission process. We grant 100% waivers to articles whose corresponding authors are based in Group A countries and 50% waivers to those who are based in Group B countries as classified by Research4Life (see: https://www.research4life.org/access/eligibility/ ). This section has its own submission website at https://mc.manuscriptcentral.com/e-videos
